# Characterization of the Dielectrophoretic Response of Different Candida Strains Using 3D Carbon Microelectrodes

**DOI:** 10.3390/mi11030255

**Published:** 2020-02-28

**Authors:** Monsur Islam, Devin Keck, Jordon Gilmore, Rodrigo Martinez-Duarte

**Affiliations:** 1 Multiscale Manufacturing Laboratory, Mechanical Engineering Department, Clemson University, Clemson, SC 29634, USA; monsur.islam@kit.edu (M.I.); dkeck@g.clemson.edu (D.K.); jagilmo@clemson.edu (J.G.); 2 Institute for Microstructure Technology, Karlsruhe Institute of Technology, Hermann-von-Helmholtz-Platz 1, 76344 Eggenstein-Leopoldshafen, Germany; 3 Department of Bioengineering, 301 Rhodes Engineering Research Center, Clemson University, Clemson, SC 29634, USA

**Keywords:** characterization, dielectrophoresis, carbon electrodes, three-dimensional (3D), diagnostics, *Candidiasis*

## Abstract

Bloodstream infection with *Candida* fungal cells remains one of the most life-threatening complications among hospitalized patients around the world. Although most of the cases are still due to *Candida albicans*, the rising incidence of infections caused by other *Candida* strains that may not respond to traditional anti-fungal treatments merits the development of a method for species-specific isolation of *Candida*. To this end, here we present the characterization of the dielectrophoresis (DEP) response of *Candida albicans*, *Candida tropicalis* and *Candida parapsilosis*. We complement such characterization with a study of the *Candida* cells morphology. The *Candida* strains exhibited subtle differences in their morphology and dimensions. All the *Candida* strains exhibited positive DEP in the range 10–500 kHz, although the strength of the DEP response was different for each *Candida* strain at different frequencies. Only *Candida tropicalis* showed positive DEP at 750 kHz. The current results show potential for manipulation and enrichment of a specific *Candida* strain at specific DEP conditions towards aiding in the rapid identification of *Candida* strains to enable the effective and timely treatment of *Candida* infections.

## 1. Introduction

*Candida* species are one of the most prevalent fungal pathogens in hospitals around the world. In the United States alone, 5%–10% of hospitalized patients will acquire a nosocomial infection and 80% of such infections are caused by *Candida* species [1]. As early as 1995, *Candida* species became recognized as the fourth most common cause of nosocomial bloodstream infections in the United States, and most recently reported as the 3rd most common cause of nosocomial bloodstream infections in the intensive care unit (ICU) [2]. Concerningly, nosocomial bloodstream infections from *Candida* have a crude mortality rate of 39% overall, and this figure can be as high as 47% for patients infected in the ICU [2]. More than 17 different *Candida* species have been identified as responsible for invasive candidiasis (IC), an umbrella term referring to various severe diseases resulting from *Candida* infection [3]. While *Candida albicans* remains the most frequently isolated *Candida* strains from infected blood [4], the incidence of the infections caused by other species has increased significantly worldwide. For example, a survey in European countries showed that around 50% infection was caused by *Candida albicans*, whereas incidence rates were 14% for each *Candida glabrata* and *Candida parapsilosis*, 7% for *Candida tropicalis* and 2% for *Candida krusei* [5]. In Chile, the most frequently isolated non-*albicans* species was *Candida parapsilosis*, followed by *Candida tropicalis* and *Candida glabrata* [6]. The emergence of non-*albicans* species as pathogens is concerning because many of them do not respond to conventional anti-fungal therapy, which are generally targeted for *Candida albicans*. For example, *Candida tropicalis* is less susceptible to fluconazole, a common anti-fungal medication, when compared to *Candida albicans* [7]. Hence, with an increased incidence of infections with different *Candida* species, there is a need for a method that allows for rapid identification of the *Candida* species, so that timely measures can be taken towards species-specific treatment of *Candida* infections.

Dielectrophoresis (DEP) is a technique that offers the potential for sorting different *Candida* species in a label-free fashion towards a rapid and affordable assay. DEP is a relatively simple procedure that works by exploiting the specific response of different cells to an electric field gradient [8,9,10,11,12], and has been used for the manipulation, separation, and enrichment of many bioparticles that include bacteria and other bloodborne pathogens [13,14,15,16,17,18,19,20] including *Candida albicans* [21,22,23,24,25,26,27]. The fact that DEP has been demonstrated in the sorting of cells featuring minor observable differences between them [9,28,29,30] encourages the study of DEP to isolate specific *Candida* strains. However, till date, no DEP characterization of *Candida* strains other than *Candida albicans* is available. Hence, there is a knowledge gap preventing the wider use of DEP as a method to sort *Candida* strains. Methodical characterization of the DEP response of *Candida* strains can enable the use of different DEP platforms towards a more rapid way to identify the type of *Candida* causing an infection and an informed approach to combat it. For example, specific *Candida* strains can be isolated and enriched from a dilute sample in a timely manner in a DEP-based sample preparation protocol previous published by the authors [8], which can increase sensitivity of common detection techniques [31].

In this work, we present the morphological characteristics and a first study on the DEP response of three different *Candida* strains: *Candida albicans*, *Candida parapsilosis*, and *Candida tropicalis*; which are three of the most frequently isolated *Candida* strains from infected samples. We used 3D carbon microelectrode arrays to obtain the results presented here due to their improved performance over more traditional planar electrodes [13,32,33].

## 2. Materials and Methods

### 2.1. Cell Culture and Sample Preparation

*Candida albicans* (ATCC 18804), *Candida parapsilosis* (ATCC22019), and *Candida tropicalis* (ATCC750) were cultured in dynamic conditions at 37 °C and 215 rpm in yeast malt broth (YMB) and passed regularly to maintain a healthy culture. To prepare the sample for DEP experiments, 100 µL of 4-day old cell culture were mixed with 2.5 mL of an optimized DEP buffer solution composed of 8.6 wt% sucrose, 0.3 wt% dextrose and 0.1 wt% bovine serum albumin to achieve a concentration of around 10^6^ cells/mL. The electrical conductivity of this DEP buffer solution was 20 µS/cm. Cells were then pelleted through centrifugation at 5000 rpm for 5 min and then resuspended into fresh DEP buffer solution. This centrifugation and re-suspension protocol were repeated three times to ensure complete removal of any remaining YMB culture media.

### 2.2. Device Fabrication

**Figure 1:**
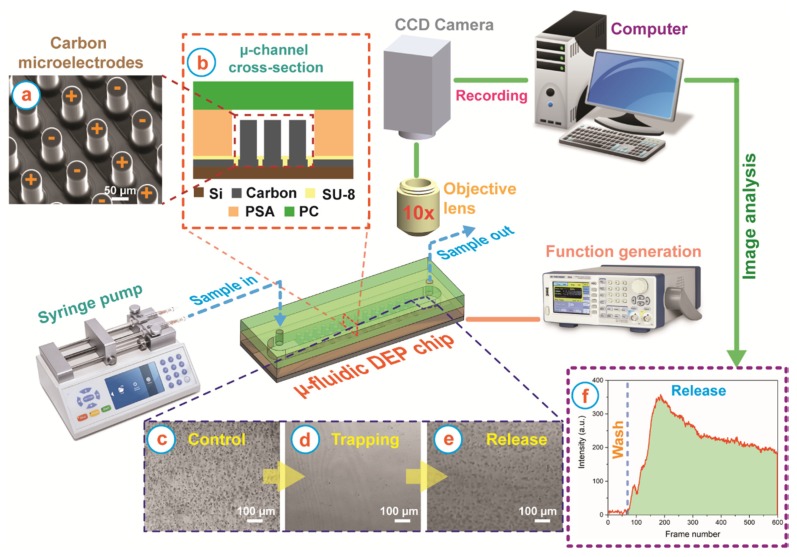
Experimental set up for the characterization of DEP response of the *Candida* strains using 3D carbon microelectrodes. (**a**) Scanning Electron Microscope (SEM) image of the 3D carbon microelectrodes. (**b**) Cross section of the microchannel showing the different elements of the DEP device. The polarity of the 3D carbon electrodes to induce the non-uniform electric field for DEP is also illustrated. The region of interest (ROI) during experiments was immediately after the last column of the electrode array. The ROI for (**c**) the control experiment (no field applied); (**d**) during the trapping stage when cells displayed a strong DEP trapping behavior; and (**e**) immediately after turning the field off to release any previously trapped cells. The black dots in these images are *Candida* cells. (**f**) An indicative plot of the normalized intensity obtained after computational analysis of the ROI throughout an experiment. The blue dashed line denotes the time when the electric field was turned off, and marks the transition between the wash and release stages. The area under the curve in the “Release” section is identified by the green area, which is reported here as the DEP trapping response of the cells in each experiment. At least three experiments were conducted for each data point, i.e., a given *Candida* strain and frequency. See text for further details.

The microfluidic DEP device used in this study featured 3D carbon microelectrode arrays. The fabrication of the carbon microelectrodes has been reported several times in our previous work [8,9,10,11,12,31,34,35,36,37]. Briefly, the fabrication process included two-step photolithography of SU-8 (Gersteltec, Switzerland), a negative tone photoresist, on a Si/SiO_2_ substrate. The SU-8 microstructures were carbonized at 900 °C in a nitrogen environment using a heating rate of 5 °C/min to obtain carbon microstructures. The resultant carbon electrode array (3161 electrodes total) featured intercalated 3D electrodes as shown in Figure 1a; each carbon microelectrode had a height of 100 µm and diameter of 50 µm while the spacing between them was around 58 µm in all directions. A thin layer of SU-8 was then patterned around the 3D carbon electrodes to insulate the connecting planar leads and planarize the channel bottom. On a parallel process, a 1.8 mm-wide and 32 mm-long channel was patterned from 127 µm-thick sheet of double sided pressure sensitive adhesive, or PSA (Switchmark 212R, Flexcon, Spencer, MA, USA), using xurography and adhered to a previously machined polycarbonate (PC) piece. The details of this method are detailed in our previous publication [38]. The DEP chip was then assembled by manually positioning the PC/PSA arrangement around the carbon microelectrode array, followed by sealing using a rolling press. The cross-section of the assembly of the microfluidic device is illustrated in Figure 1b.

### 2.3. Experimental Protocol

Experiments revolved around characterizing: (1) cell morphology and (2) DEP response. Few studies are available regarding the morphological characterization of few *Candida* strains [39,40] and a morphology study was performed here to better understand how the unique morphology of the cells from each species may contribute to a difference in their response to an electric field. To this end, 4-day cell cultures were observed under an optical microscope (Nikon Eclipse LV100, Tokyo, Japan) to measure the dimensions of at least 30 cells per strain. Images were recorded through an Andor Zyla CMOS camera.

The experimental set up for characterization of the DEP response of the cells at different frequencies is illustrated in Figure 1. The experimental protocol followed can be separated into 3 stages: (1) cell trapping, (2) washing, and (3) cell release. Of note, cell release only occurred in the frequencies that lead to cell trapping due to positive DEP: this is when the cells were attracted to the regions of high field gradient that are around the carbon electrodes in this work. The desired flow rate in the experimental device was implemented using a syringe pump (FusionTouch 200, Chemyx, Stafford, TX, USA). The electrode array was polarized as illustrated in Figure 1a using a function generator (BK Precision 4052, Yorba Linda, CA, USA). During the first experimental stage, trapping, 20 µL of the sample containing the cells was flowed at 10 µL/min through the electrode array polarized at specific frequency (10 kHz–1 MHz) and magnitude of 20 V_pp_. In the second stage, washing, a cell-free DEP buffer solution was flowed with a flow rate of 10 µL/min through the still polarized electrode array for 5 min to wash any non-trapped cells. In the last stage, release, the polarizing signal was turned off while maintaining the same flow rate. This last stage lasted for 110 s. The entire experiment was monitored through a 10× objective lens in a Nikon Eclipse LV100 microscope. However, only the release stage and the last 10 s of the wash stage were recorded using an Andor Zyla CMOS camera running at a frame rate of 5 frames per second. Hence, each of the video recordings used for data analysis was 120 s, or 600 frames, long. The electric field was turned off at frame 50.

### 2.4. Data Analysis

Images of cell cultures were manually analyzed in the NIS Element Basic software native to the microscope to measure the major (*x*) and minor axes (*y*) of at least 30 cells per strain. The major and minor axes were identical for a perfect circle. The average values and standard deviation for all measurement were calculated using built-in mathematical functions in Microsoft Excel.

The videos obtained during DEP experiments were analyzed with ImageJ (National Institutes of Health, Bethesda, MD, USA) to plot the average pixel intensity at a region of interest (ROI), with area 830 µm × 700 µm and established immediately after the electrode array, throughout each experiment done for a particular cell strain and frequency of interest (Figure 1c–e). The analysis was designed such that a difference in the intensity of the ROI before and after turning the field off can be directly correlated to the strength of the trapping DEP force acting on a given sample at that specific frequency. To this end, the average intensity in the ROI was measured for a total of 600 frames, where frames 1–50 were for the frames recorded before turning the field off and frame 51–600 were recorded after the field was turned off. In order to properly isolate the DEP response from each experiment, all the intensity values after turning the field off were normalized against the average intensity before the field was off. These normalized values were then plotted using Origin Pro software (OriginPro 2016, Northampton, MA, USA). An example of such plot is shown in Figure 1f for an experiment that exhibited cell trapping and release. The larger the curve would denote a larger number of cells trapped, and released, and thus a stronger DEP trapping response. No DEP trapping resulted in no curve, i.e., a flat line after turning the field off. Here, we report the area under the curve in frames 51–600 to represent the DEP trapping response of the *Candida* strains.

## 3. Results

### 3.1. Morphology of the Candida Strains

**Figure 2:**
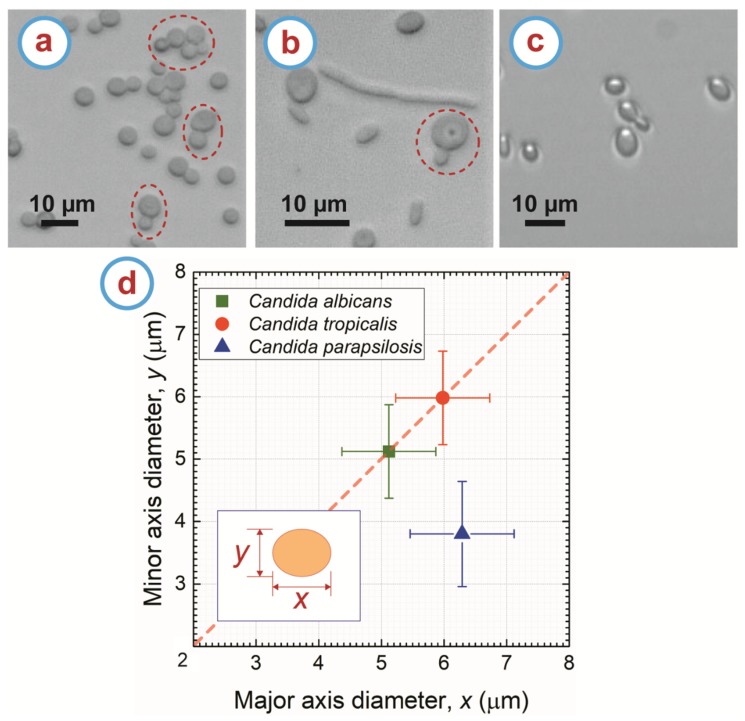
Morphology of (**a**) Candida albicans, (**b**) Candida tropicalis, and (**c**) Candida parapsilosis. The dashed circles indicate the budding behavior of the cells. (**d**) Plot of the major (*x*) vs minor diameter (*y*) of the different Candida strains. The diameters in the major and minor axes of a cell are illustrated in the inset. For a spherical cell, *x* is identical to y as denoted by the red dashed line. Each data point represents the average from at least 30 cells for each strain. The error bar represents the standard deviation from all measurements.

Candida albicans (Figure 2a) displayed an average spherical morphology, which means the major axis diameter (*x*) of Candida albicans is identical to the minor axis diameter (*y*). Candida albicans featured an average diameter of *x* = *y* = 5.12 ± 0.75 µm, as depicted in Figure 2d. In addition to their largely spherical morphology, Candida albicans were commonly found in their budding phase of reproduction offering an alternate morphology of two or more attached spheres, as indicated by the dashed circles in Figure 2a. Such morphologies of the Candida albicans cells are in agreement with previous reports [39,40].

Candida tropicalis exhibited multiple morphologies. The first was a spherical morphology similar to that of Candida albicans but with larger diameter of *x* = *y* = 5.98 ± 0.75 µm, which is consistent with previous findings by other authors [39]. As in the case for Candida albicans, Candida tropicalis also displayed spherical morphology in its budding phase of reproduction. The second shape is referred to as pseudohyphae [39,41,42] and resulted when cells began to bud but instead of separating the membranes of the cell merged to become one elongated cell. This happened multiple times in our observations, leading the pseudohyphae to have elongated ellipsoidal morphology and even beginning to resemble a tree when multiple branches of elongated ellipsoids formed. The length of the pseudohyphae ranged from 7 µm to 27 µm with an average width of 1.89 ± 0.4 µm as seen in Figure 2b.

Candida parapsilosis shown in Figure 2c displayed an ellipsoidal morphology. The diameters of each of the two axes were found to be *x* = 6.29 ± 0.83 µm and *y* = 3.8 ± 0.84 µm, and these values are in the range of the dimensions reported elsewhere for this strain [39]. Although less frequent than in *Candida albicans, Candida parapsilosis* was also found in its budding stage but with a featuring morphology that resembled two or more attached ellipsoids instead of spheres.

### 3.2. Trap, Wash and Release of Candida Cells

**Figure 3:**
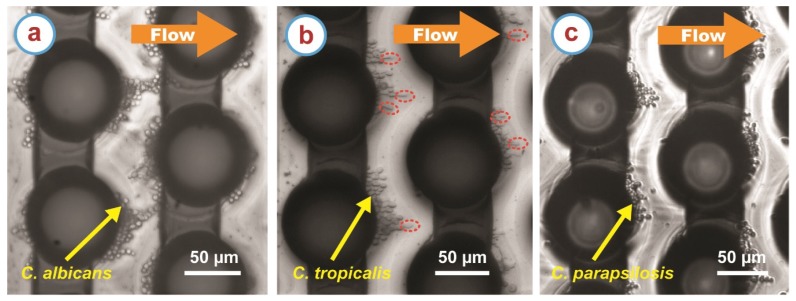
Examples of trapping of (**a**) *Candida albicans*, (**b**) *Candida tropicalis*, and (**c**) *Candida parapsilosis* cells on the carbon microelectrodes (the dark circles and connecting lines) due to positive dielectrophoresis. These specific examples are when the frequency of the applied electric field is 100 kHz. Note that for *Candida tropicalis*, few short length pseudohyphae cells were also trapped and indicated with the red dotted ellipse in (b).

We were able to trap, wash and release different *Candida* strains at will. Upon applying the electric field at specific frequencies, the *Candida* cells experienced positiveDEP force and got trapped in the regions of high field gradient around the carbon microelectrodes (Figure 3). Our DEP device has a capacity of trapping around 4000 cells as previously reported [8]. In our DEP experiments, the microchannel was loaded with an experimental sample featuring a cell concentration of 10^6^ cells/mL, which translates to a total number of cells ~7000 present in the microchannel to start with. Hence, upon applying the electric field, our device could trap the *Candida* cells to its capacity, and the wash protocol ensured carrying away the untrapped cells, enabling purification of the trapped cells. Due to the cell trapping, no cells were seen in the flow in the recording area at the end of the electrode array before the cell release (Figure 1d). Upon turning off the electric field, the trapped cells on the electrodes were eluted through the recording area (Figure 1e). Of note, a small number of cells was observed to be non-specifically adhered to the electrodes after turning off the polarizing signal. The number of such cells was significantly smaller than those released. Furthermore, this non-specific adhesion was observed for all strains studied in this work. Hence, the effect of such adhesion on the characterization of the DEP response for the different strains, and the differences between them, was deemed not significant. This non-specific adhesion may be detrimental to future assays where the recovery of targeted cells would be required but this out of the scope of the work presented here.

### 3.3. DEP Response of the Candida Strains

**Figure 4:**
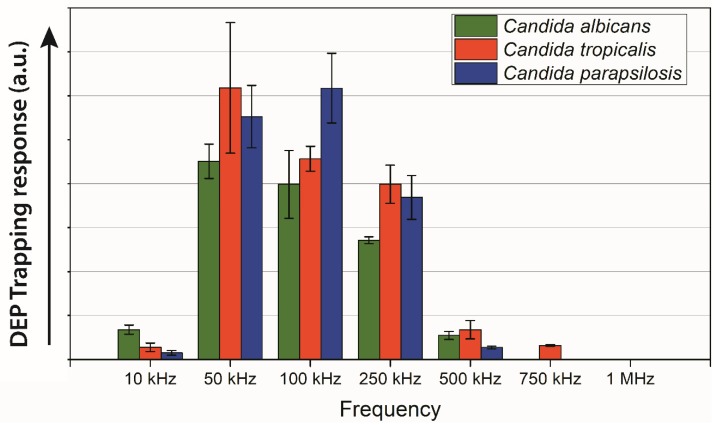
The DEP trapping response of *Candida albicans*, *Candida tropicalis*, and *Candida parapsilosis* at frequencies ranging from 10 kHz to 1 MHz. At least three experiments were carried out for each data point, bars denote the average values while the bars represent standard deviation. The upward arrow indicates that the higher value represents higher cell trapping due to DEP.

The DEP trapping response for the different *Candida* species characterized in this work is plotted in Figure 4. *Candida albicans* and *Candida parapsilosis* exhibited a trapping DEP response in the frequency range 10–500 kHz. *Candida tropicalis* also showed a trapping positive DEP response at 750 kHz. No positive DEP was observed beyond 750 kHz for any of the *Candida* strains. Although all the three strains showed positive DEP response in the range 10–500 kHz, the strength of the DEP response was different for each strain. The highest DEP trapping response for *Candida tropicalis* and *Candida albicans* was at 50 kHz, whereas *Candida parapsilosis* showed the peak DEP response at 100 kHz. *Candida albicans* exhibited relatively weak DEP trapping compared to the other two strains of *Candida* in the frequency range 50–500 kHz. However, at 10 kHz, the positive DEP response was strongest for *Candida albicans* among the three *Candida* strains.

## 4. Discussion

The *Candida* strains studied here showed subtle differences in cell morphologies. Figure 2d shows how the cells dimensions overlap for all the strains, making it difficult to sort *Candida* strains solely based on their sizes. The dimensional overlap is more prevalent when comparing *Candida albicans* and *Candida tropicalis*. Moreover, both *Candida albicans* and *Candida tropicalis* exhibited spherical morphology, further complicating differentiation based on shape. Although pseudohyphae forms of *Candida tropicalis* exhibits different shape than the spherical cells, *Candida albicans* is also known to transform to hyphal state during infectious process [43,44,45] and this must be taken into account even when we did not observe hyphal forms in our handling of *C. albicans*. Although size and shape are not enough for cell separation, these can be complemented with the DEP response of specific strains.

All the *Candida* strains exhibited positive DEP response in the frequency range 10–500 kHz. However, *Candida tropicalis* and *Candida parapsilosis* consistently exhibited higher positive DEP response than *Candida albicans* in the frequency range 50–250 kHz. Such behavior might be a result of the budding behavior of *Candida albicans*. *Candida albicans* were more commonly found in their budding stage as a group of multiple attached spheres. The drag force created by a conglomeration of attached spherical entities would have a higher magnitude than the drag force of a smaller spherical entity [46]. If the drag force becomes larger than the DEP force created from the electrical field gradient, the cells would not attract to the electrodes [30]. However, at 10 kHz, different DEP behavior was observed, where *Candida albicans* exhibited strongest DEP response. Our hypothesis is that different *Candida* strains might feature different cell membrane potential, which resulted in different behaviors in different frequency range. However, the cell membrane potentials for *Candida* strains are unknown at present. A separate, more extensive study to determine the cell membrane potential of these *Candida* strains is ongoing and will be reported in a future work.

*Candida tropicalis* shows both spherical and pseudohyphae morphology in the media studied here as shown in Figure 2b. However, mostly spherical *Candida tropicalis* cells were observed on the carbon microelectrodes during trapping (Figure 3b). A small amount of short ranged pseudohyphae cells with length ranging from 7 µm to 12 µm were also trapped on the carbon electrodes. No long range pseudohyphae cells were trapped on the carbon microelectrodes. We speculate that as the *Candida tropicalis* cells transforms to pseudohyphae cells, the cell membrane potential might also change. The difference in the cell membrane potential of the spherical and pseudohyphae cells might be in direct proportion of the length of the pseudohyphae cells. For example, the short pseudohyphae cells might feature a cell membrane potential close to that of the spherical cells. This may lead to trapping of short length pseudohyphae cells in the current DEP conditions along with the spherical cells, whereas the long pseudohyphae cells did not experience any positive DEP force and flow with media during the washing step.

The current results indicate a potential for DEP to be utilized to distinguish between different types of *Candida* strains from an already purified blood sample and help diagnose the specific strain causing disease. One of the important results in this direction is that *Candida tropicalis* was the only *Candida* strain to show a positive response at 750 kHz. In terms of strain identification, a sample could be subjected to an electric field with frequency of 750 kHz to only trap *C. tropicalis* while eluting *Candida albicans* and *Candida parapsilosis*. Further separation between *C. albicans* and *C. parapsilosis* could be done in a second stage, i.e., polarized at 10 kHz to emphasize trapping of *C. albicans*, in a multi-stage carbon-electrode DEP device as previously reported [47]. Furthermore, each *Candida* strain exhibited a difference in the strength of the positive DEP response, which can be also utilized for diagnosis purposes. This characteristic could be used in multiple ways. One potential method would be to tailor the strength of the electric field within the DEP device to be strong enough to attract one type of cell, but too weak to trap another *Candida* strains allowing these *Candida* strains to be eliminated as the cause of infection. The trap and wash protocol demonstrated here can be used to enrich desired *Candida* cells in a small sample volume for further analysis. Using the current DEP set up, it is possible to enrich a cell sample up to 150 folds within a few hours as we previously reported [8]. This rich enrichment can lead to a timely detection of the *Candida* cells by enabling concentrated and purified samples to improve the sensitivity of common detection protocols such as PCR [31]. Another approach might be using streaming DEP for rapid cell sorting, where cells are focused into specific streams of elution instead of trapping cells on the electrodes. Streaming DEP can enable focusing of different *Candida* cells into different streams utilizing the different strength of DEP on the different *Candida* strains [11,30]. The streams can be collected separately at the outlet of the microfluidic system and used for cell detection. Such timely detection of the *Candida* cells can enable timely initiation of medical treatment specific to the responsible *Candida* strains.

It should be noted that the value of DEP in a practical solution for rapid diagnosis of *Candida* infection is envisioned to be the isolation of different strains of *Candida* from each other, but not directly from blood. A multi-stage protocol is preferred when attempting to isolate potential targets of interest from a blood sample in clinical diagnostics. For example, centrifugation can enable a first rapid coarse separation of serum, buffy coat and red blood cells (RBC) [48], with the *Candida* cells expected to be in the buffy coat [49]. Buffer exchange protocols common in clinical diagnostics can then be implemented to re-suspend the cellular content of the buffy coat in a buffer optimized for DEP and any other downstream processing. Further stages in the process can include size exclusion to further isolate *Candida* cells from other blood cells until only particles that may resemble *Candida* are present in the sample. At this stage, a DEP assay for fine separation could be used to perform isolation and purification of specific strains to increase the performance of detection assays such as PCR as detailed above. If necessary, the DEP properties of blood cells with similar sizes than *Candida* have been characterized [9,50,51] and such knowledge could be used to aid in their separation. Such a multi-stage process can be readily implemented in a clinical setting. However, the details of such integrated assay are out of the scope of this paper. Of note, the integration of DEP with centrifugal microfluidics towards enabling such integrated assay has been reported by one of us [13,35].

## 5. Conclusions

The characterization of the morphology and DEP response of three frequently isolated *Candida* strains from infected samples: *Candida albicans*, *Candida tropicalis*, and *Candida parapsilosis* was presented here. The studied *Candida* strains only exhibited subtle differences in morphology that makes direct observation an incomplete method to sort them. 3D carbon electrode DEP was implemented for characterizing the DEP response of the *Candida* cells. All three *Candida* strains showed a strong positive DEP response in the frequency range 10–500 kHz. However, positive DEP at 750 kHz was only observed for *Candida tropicalis*. Furthermore, the *Candida* strains exhibited the positive DEP at different strength. *Candida tropicalis* and *Candida parapsilosis* showed relative high DEP response than *Candida albicans* in the frequency range 50–250 kHz, whereas at 10 kHz, the DEP response was strongest for *Candida albicans*. Together, the morphological and DEP differences among the different strains could provide a framework to enable sorting different strains.

This is to the best of our knowledge the first study reporting the DEP responses of different *Candida* strains. The current results show promise towards using DEP as a tool to enable separation of different *Candida* species. Ongoing work focuses on characterizing the membrane capacitance of the strains presented here and expanding this study to other relevant strains such as *Candida glabrata* and *Candida krusei*. The results presented here indicate that one can potentially manipulate and enrich a specific *Candida* strain at specific DEP conditions and encourages further work towards rapid identification to the enable effective and timely treatment of candidiasis.
